# Telomere Length in Elderly Caucasians Weakly Correlates with Blood Cell Counts

**DOI:** 10.1155/2013/153608

**Published:** 2013-12-21

**Authors:** Ewa Gutmajster, Joanna Witecka, Magdalena Wyskida, Justyna Koscinska-Marczewska, Malgorzata Szwed, Magdalena Owczarz, Malgorzata Mossakowska, Andrzej Milewicz, Monika Puzianowska-Kuznicka, Jan Zejda, Andrzej Wiecek, Jerzy Chudek, Aleksander L. Sieron

**Affiliations:** ^1^Department of General and Molecular Biology and Genetics, Medical University of Silesia, 40-752 Katowice, Poland; ^2^Department of Pathophysiology, Medical University of Silesia, 40-752 Katowice, Poland; ^3^Department of Human Epigenetics, Mossakowski Medical Research Centre, 02-106 Warsaw, Poland; ^4^International Institute of Molecular and Cell Biology, 02-109 Warsaw, Poland; ^5^Department of Endocrinology, Diabetology and Isotope Therapy Medical University, 50-367 Wroclaw, Poland; ^6^Department of Geriatrics and Gerontology, Medical Center of Postgraduate Education, 01-813 Warsaw, Poland; ^7^Department of Epidemiology, Medical University of Silesia, 40-752 Katowice, Poland; ^8^Department of Nephrology, Endocrinology and Metabolic Diseases, Medical University of Silesia, 40-027 Katowice, Poland

## Abstract

*Background*. Age-related decrease in bone marrow erythropoietic capacity is often accompanied by the telomere length shortening in peripheral white blood cells. However, limited and conflicting data hamper the conclusive opinion regarding this relationship. Therefore, the aim of this study was to assess an association between telomere length and peripheral blood cell count parameters in the Polish elderly population. *Material and Methods*. The substudy included 1573 of 4981 subjects aged 65 years or over, participants of the population-based PolSenior study. High-molecular-weight DNA was isolated from blood mononuclear cells. Telomere length (TL) was measured by QRT-PCR as abundance of telomere template versus a single gene copy encoding acidic ribosomal phosphoprotein P0. *Results*. Only white blood count (WBC) was significantly different in TL tertile subgroups in all subjects (*P* = 0.02) and in men (*P* = 0.01), but not in women. Merely in men significant but weak positive correlations were found between TL and WBC (*r* = 0.11, *P* < 0.05) and RBC (*r* = 0.08, *P* < 0.05). The multiple regression analysis models confirmed a weak, independent contribution of TL to both RBC and WBC. *Conclusions*. In the elderly, telomere shortening limits hematopoiesis capacity to a very limited extent.

## 1. Introduction

The process of bone marrow senescence involves gradual replacement of hematopoietic tissue by adipocytes and fibroblasts [1]. This process is particularly evident in people aged over 80 years, when the hematopoietic tissue mass is reduced by half compared with young adults [2].

Bone marrow senescence results in the decreased number of erythroblasts, which is partially related to the age-associated reduction of erythropoietin (EPO) production by the kidneys and to the high prevalence of chronic kidney disease in the elderly [3, 4]. EPO prevents apoptosis of red cell progenitors and stimulates the synthesis of heme and hemoglobin as well as iron incorporation by erythroblasts. Important role in the aging of the hematopoietic system is attributed to low androgen levels both in men and in women, that occurs after menopause or andropause [5]. In addition, frequent deficiencies of iron, vitamin B_12_, folic acid, and of other cofactors of hematopoiesis in the elderly are attributed to poor diet regardless of coexisting comorbidities [6].

Bone marrow senescence is also related to the accumulation of DNA damage in hematopoietic stem cells (HSCs) [7]. Although it was suggested that telomerase activity in HSCs should maintain a stable telomere length (TL) [8], emerging evidence demonstrated that telomere shortening can limit the maintenance and function of the adult stem cells [9]. Probably, the telomerase activity in HSCs is inadequate to prevent telomere erosion and, therefore, unable to avoid cell aging [10–12]. However, Wang et al. have recently shown that the telomerase activity varied in HSCs from very weak to the levels comparable to these seen in the immortal cell lines, with no apparent age-dependent decline [13].

WBC TL can be used as an approximate for HSCs TL, therefore, most probably, it can be also used as an estimate for nucleated blood cells progenitors TL [14, 15]. The association between WBC TL and peripheral blood count parameters was studied among middle-aged [16], mix middle-aged and elderly [17], and elderly [18] populations. The results of these studies are inconsistent. De Meyer et al., who studied a large cohort (*n* = 2508) of 35–55 years old population, found leukocyte TL to be predictive of red blood cell (RBC) count [16]. This finding is in contrast to the finding reported by Mollica et al. who did not observe any significant correlation in a large cohort (*n* = 717) of women aged 38–100 years [17]. Den Elzen et al. compared TL between participants aged 85 and over with and without anaemia (*n* = 349 and *n* = 1058, resp.) showing no correlation between leukocyte TL and haemoglobin concentration and other haematological parameters [18]. These published evidence is not decisive and the unsolved issue justifies further studies in that field. We have decided to investigate the effect of TL on peripheral blood cell counts in a sample of population-based PolSenior study [19]. The aim of the study was to assess an association between telomere length and peripheral blood cell count parameters in the Polish elderly population.

## 2. Material and Methods

### 2.1. Study Design and Setting

PolSenior was a multicenter, interdisciplinary project, conducted in the years 2007–2011, designed to assess health and socioeconomic status of the elderly population in Poland. The study design was described elsewhere [19]. The study included comprehensive geriatric assessment based on standardized questionnaire and collection of blood samples.

### 2.2. Material

4979 elderly subjects agreed to participate in a questionnaire survey (42.6% of all eligible subjects). Peripheral blood analysis was performed in 4003 subjects. First 1573 of approximately 4000 genomic DNA isolated from blood mononuclear cells were included into the study. Characteristics of this group (31.6% of the whole study population) are shown in Table 1.

### 2.3. Methods

#### 2.3.1. DNA Isolation

Four ml blood samples were used for the isolation of the genomic DNA by salting-out procedure [20]. The concentration and purity of DNA were assessed by UV spectroscopy (Nanodrop, Thermo Fisher Scientific Inc., Wilmington, DE, USA).

#### 2.3.2. Telomere Lengths (TL) Assay

WBC TL was measured using the real-time quantitative polymerase chain reaction (PCR) method. This method determines, for each DNA sample, the factor by which it differs from a reference DNA sample in its ratio of telomere repeat copy number to a single copy gene copy number. Using two primer pairs that target telomeric hexamer repeats and a single copy gene (*36B4*, which encodes acidic ribosomal phosphoprotein P0), the telomere repeat copy number (T) to single copy gene copy number (S) ratio was calculated for each DNA sample. The T/S ratio of each experimental DNA sample was related to a reference DNA sample (obtained from a single individual and used for standard curve generation) of which T/S ratio is 1. The forward and reverse primer sequences were for telomeres amplification: 5′CGG TTT GTT TGG GTT TGG GTT TGG GTT TGG GTT TGG GTT 3′ (39 bp) and: 5′GGC TTG CCT TAC CCT TAC CCT TAC CCT TAC CCT TAC CCT 3′ (39 bp), respectively [21]. For the acidic ribosomal phosphoprotein P0 (*36B4*), the forward and reverse primer sequences were 5′CAG CAA GTG GGA AGG TGT AAT CC 3′ (23 bp) and 5′CCC ATT CTA TCATCA ACG GGT ACA A 3′ (25 bp), respectively [22]. For PCR amplification, reactions were incubated for 5 min at 95°C and then amplified over 45 cycles of 10 s at 95°C, 10 s at 55°C and 30 s at 72°C. Reactions were conducted in LIGHT CYCLER 480 (Roche, Germany). All samples were processed in triplicate. Telomere length was reflected by the relative telomere/single copy gene ratio (T/S) values: T/S = 2^−ΔCt^ (ΔCt = Ct^telomere^ − Ct^36B4^).

The T/S ratio for each participant was measured in triplicates. When the T/S values varied by more than 20%, the sample was run for a second time, again in triplicates. The result with lower standard error was chosen for further calculations. The intraassay reproducibility was calculated as 10.4%.

To asses interassay reproducibility the same control DNA (Human Genomic DNA, ROCHE, Germany) was used in each plate run. The average standard deviations for a single-copy gene did not exceed 3.6%.

#### 2.3.3. Laboratory Measurements

Serum iron, uric acid, creatinine, and C-reactive protein (by a high sensitivity method) levels and peripheral blood cells levels were measured in the central laboratory of the PolSenior study (on Modular PPE analyzer, Roche Diagnostics GMbH, Mannheim, Germany). Folic acid and vitamin B_12_ (by RIA method, DIAsource ImmunoAssays, Nivelles, Belgium), 25-OH-D_3_ (by RIA method, DIAsource ImmunoAssays, Nivelles, Belgium), ferritin (by ECLIA method for Cobas e411, Roche Diagnostics GMbH, Mannheim, Germany), and testosterone (by RIA method, Siemens, Los Angeles, USA) were assessed in Laboratories of the Department of Nephrology Endocrinology and Metabolic Diseases Medical University of Silesia in Katowice and of the Department of Endocrinology, Diabetology and Isotope Therapy Medical University in Wroclaw.

#### 2.3.4. Data Analysis

Data analysis involved above-mentioned measurements and questionnaire-derived data on gender, age, body mass index (BMI), and smoking status. Apart from gender and smoking status, all variables were analyzed as quantitative variables. Distribution of quantitative variables was shown by mean values and standard deviations. Because of abnormal distributions (result of Shapiro-Wilk test), the between-group differences in quantitative variables distributions were examined by Kruskal-Wallis test.

Effect of TL on peripheral blood cells levels was explored by the means of univariate and multivariate analyses. Univariate analyses involved the comparison of mean values between three classes of TL, defined by subsequent tertiles of the total and gender-specific distributions (0–33.3%; 33.4–66.6%; 66.7–100%). Moreover, Spearman correlation coefficients and their *P* values were calculated to measure association between TL and peripheral blood cell levels.

In multivariate analyses the effect of TL on peripheral blood measurements was examined using linear logistic regression. Three separate models included RBC, WB, and PLT as dependent variables. In each model, the variable expressing TL was entered after logarithmic transformation to improve linearity of that variable. In each model, the effect of TL on RBC, WB, and on PLT was analyzed after adjustment for age, gender, vit. B_12_, folic acid, iron, CRP, testosterone, and eGFR levels. The set of independent variables was examined for a potential effect of multicollinearity using analysis of correlation. Only variables that were involved in associations below *r* < 0.5 were entered in the model.

Statistical analyses were performed using SAS software version 9.1.3 (SAS Institute Inc., Cary, NC) and statistical inference was based on the criterion *P* < 0.05, both for univariate and multivariate analyses.

## 3. Results

### 3.1. Characteristics of Study Population

Among 1573 participants with determined TL there were 810 men and 763 women. This group was representative for the PolSenior study population (Table 1). There was a small, but statistically significant difference in age between women with determined and notdetermined TL (*P* < 0.001). No such differences were detected in the whole group and in men (*P* > 0.05). Two hundred seventeen study participants were permanently using nonsteroidal anti-inflammatory drugs (NSAIDs), well known myelotoxic drugs.

### 3.2. TL Effect on Total Blood Cell Count: Results of Univariate Analyses

TL was highly variable (mean value ± SD 106.4 ± 110.8, median 72.2, and kurtosis 16.2). After logarithmic transformation, the mean value of logTL was 4.23 ± 0.96 (median 4.26 and kurtosis −0.04). A tendency for shorter logTL values was observed in men compared to women (4.19 ± 0.95 versus 4.28 ± 0.98; *P* = 0.06). There was no significant correlation between logTL and age.

**Figure 1:**
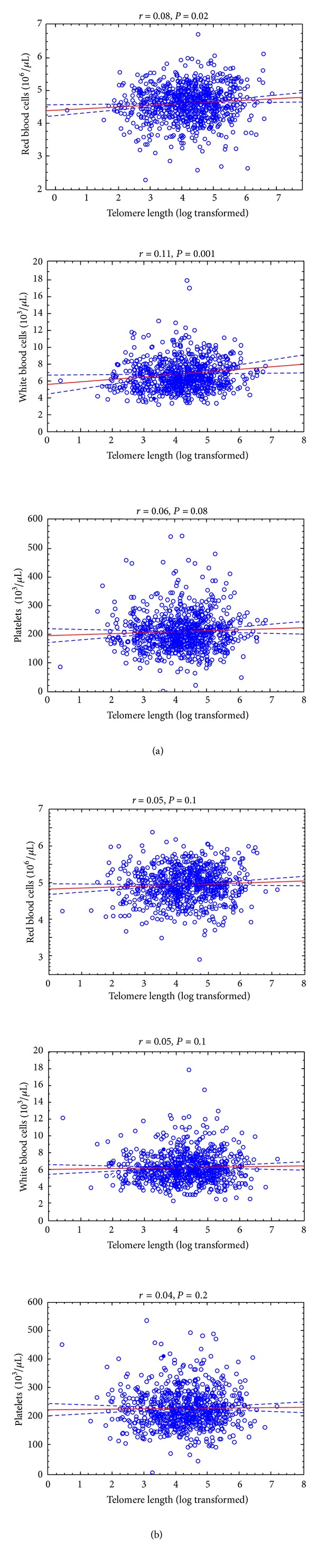
Correlation between telomere length and peripheral blood count (red blood cell, white blood cell, and platelets) in men (a) and women (b).

Data shown in Table 2 suggest a trend towards higher counts of some blood cell types with increasing TL. However, statistically significant differences were found only in relation to WBC in all subjects (*P* = 0.02) and in men (*P* = 0.01), but not in women (*P* > 0.05). Moreover, in women the highest counts of lymphocytes and monocytes were found in the second tertile category of TL (*P* < 0.05). There were statistically significant, but weak correlations between TL and WBC (*r* = 0.08), RBC (*r* = 0.06), and PLT (*r* = 0.06) in all subjects. These correlations were even stronger in men for RBC (*r* = 0.08) and WBC (*r* = 0.11) (Figure 1). The correlation with PLT was almost statistically significant (Table 3). No correlation was found in women.

There was no correlation between circulating levels of vitamin B_12_, folic acid, iron, ferritin, CRP, IL-6, and 25-OH-D_3_ and TL in all study participants together and in each gender separately.

### 3.3. TL and Total Blood Cell Count: Results of Multivariate Analyses

The effect of TL on total blood cells counts (RBC, WBC, and PLT) was examined by means of multivariable linear regression analysis. The models included the following variables: age, gender, the levels of vitamin B_12_, folic acid, iron, CRP, testosterone, eGFR, and TL. Because of its skewed distribution, the last variable was entered in the model after logarithmic transformation.

In order to avoid the effect of multicollinearity, all independent variables were examined for between-variable associations. The largest correlations were seen between age and eGFR (*r* = −0.44) and between serum levels of iron and CRP (*r* = −0.30). All remaining correlation coefficients did not exceed 0.1. Based on the above results, all of the independent variables were retained in the complete model including 1264 observations. Results of multivariable analyses (Table 3) showed that, after multiple adjustment, both RBC and WBC were significantly related to the telomere length (*β* = 0.029, *P* = 0.03 and *β* = 0.154, *P* = 0.01, resp.). However, TL did not explain the levels of PLT in a statistically significant way (*P* = 0.3). Moreover, the results of multiple regression models demonstrated that age-related decrease of the RBC, WBC, and PLT cannot be explained only by TL shortening, declining eGFR, inflammation, decreasing testosterone level and systemic inflammation, iron and folic acid, and vitamin B_12_ deficiency (Table 4).

## 4. Discussion

An association between TL and decreased bone marrow proliferation capacity in elderly is controversial. Simple analyses indicate impaired hematopoietic potential of HSCs; however, microelements and vitamins deficiency (iron, vitamin B_6_, B_12_, and folic acid), systemic inflammation, and other factors have also impact on this process. Having this in mind, we have tested the association between TL and blood cell counts (RBC, WBC, and platelets) and other parameters (hemoglobin and MCV).

Our study confirmed that high variability of TL [23] weakens the significance of gender-specific differences in elderly. Because of this, we performed a separate analyses of TL tertiles of the total and gender-specific distributions. Only in men intertertile difference for WBC was demonstrated. In univariate analyses, WBC and RBC were proportional to TL exclusively in men. These relations were confirmed in multivariate regression analyses showing that TL is an independent explanatory variable for RBC and WBC counts in men but not in women. Previously, De Meyer et al. showed such an association for RBC, but not for WBC, both in men and women of middle age [16]. Two other studies failed to prove significant correlation between TL and blood cell parameters [17, 18]. However, the study of Mollica et al. included only women in wide range of age (dispersion of age: 38–100) [17], while the study of Den Elzen et al. analyzed the population of 85+ seniors which is more comparable to the PolSenior study [18]. They failed to prove the association between TL and hemoglobin concentration, hematocrit, MCV, RBC, PLT, and WBC in these very old subjects. Most possibly, the weak association between TL and hematological parameters was blurred by other factors. Generally shorter TL in men may explain gender-specific differences in the degree of peripheral blood cell count. Though in comparison to women, it should be easier to detect correlations in men, supporting the reported associations in this population. Another possible explanation for the lack of association between TL and WBC count in women may be potential protective influence of hormones in their earlier life [24].

The results of multiple regression models demonstrate that age-related decrease of RBC, WBC, and PLT counts cannot be explained only by TL shortening, declining eGFR, inflammation, decreasing testosterone level and systemic inflammation, and iron and vitamins deficiency. Therefore the existence of other not analysed age-associated factors should be taken into consideration, such as accumulation of DNA damage that decreases proliferation potential of HSCs. Defects in stem cell self-renewal and in the maintenance of proliferating tissues that have been associated with decreased expression of telomerase caused by gene mutations are clinically relevant approximately till the age of 40 [25], whereas in the elderly external factors (xenobiotics, toxins, inappropriate diet and unhealthy lifestyle, and infections) and chronic inflammation have dominant impact on age-related nontelomeric DNA damage, which is especially marked in tissues with high rates of cell turnover (HSCs, skin, and intestine epithelia). These factors may alter the functional capacity of HSCs in repair double-strand breaks, thereby deteriorating an important genome protection mechanism leading to exceeding DNA damage accumulation [7].

The results of our study suggest rather limited role of genetic background for age-related decrease of bone marrow proliferation capacity. In mathematical models, telomere shortening explains hematopoietic bone marrow senescence only to a small extent. Moreover, telomere shortening does not explain the declining hemoglobin levels. It was shown that decrease in EPO response to low hemoglobin concentrations contributes to anemia development in the elderly [26]. On the other hand, EPO levels were reported to increase in healthy aging, at least in men [26], suggesting the increasing resistance of erythroid progenitor cells to this stimulating factor. In theory, increased EPO levels, at least in patients suffering from deficient anemia, by promoting telomerase activity in erythroid progenitor cells, should support the relevance of TL maintenance. However, Den Elzen et al. found no differences in median TLs between participants with anemia and those without anemia [18]. Similarly, in our study no association between TL and hemoglobin level was found. These results support the hypothesis that telomerase activity stimulated by EPO is insufficient to avoid net telomere loss, probably resulting in replicative senescence.

It should be emphasized that the TL shortening translates to the decreased RBC count but not to the lower hemoglobin concentration or to the lower volume of erythrocytes (MCV). This observation suggests that TL attrition restricts proliferative bone marrow potential to a limited extent but does not impair hemoglobin synthesis in erythroblasts. Thus, the process of TL shortening most possibly does not result in the development of anemia, at least in elderly.

One of the limitations of our study was that we did not examine TL in HSCs but in WBC and this was assumed to reflect bone marrow senescence status. Population-based study incorporating bone marrow biopsies, however, interesting from the scientific point of view, is not realistic [27]. Moreover, it has to be emphasized that several conditions such as oxidative stress or systemic microinflammation can influence telomere attrition in WBC. However, we did not find any association between TL and the levels of circulating CRP and IL-6. The parameters of oxidative stress were assessed only in some respondents, therefore we did not include them in the analysis. Additionally we did not examine the associations of the telomerase reverse transcriptase single nucleotide polymorphisms with TL which will be an issue for the further study of PolSenior population. A strength of our analysis is that we gathered data from a large population-based study of individuals aged 65 years and over, allowing us to generalize our results to the population of elderly people at large.

In conclusion, our study showed that TL shortening may be associated with decreased hematological parameters in older individuals; however, TL contributes to such a decrease in a very limited extent. Nevertheless, this observation supports the hypothesis that shorter HSCs telomeres might limit hematopoesis capacity.

## Figures and Tables

**Table 1:** The characteristics of study group and its comparison with the group without assessment of telomere lengths and peripheral blood count.

	Study group		Group not included		Statistical significance	
	Men	Women	Men	Women	Men versus men	Women versus women
	(*N* = 810)	(*N* = 763)	(*N* = 1756)	(*N* = 1649)		
Age (years)	79.2 ± 8.5	78.1 ± 8.6	79.6 ± 8.6	79.7 ± 8.9	Ns	*P* < 0.001
BMI (kg/m^2^)	27.3 ± 4.5	29.1 ± 5.4	27.4 ± 4.5	28.8 ± 5.6	Ns	Ns
Smoking status (*n* (%))						
Never	258 (31.9)	610 (79.9)	579 (33.0)	1354 (82.1)	Ns	Ns
Former	551 (68.0)	148 (19.4)	1169 (66.6)	282 (17.1)	Ns	Ns
Active	118 (14.6)	38 (5.0)	228 (13.0)	65 (3.9)	Ns	Ns
Pack-years	34.5 ± 26.0	21.8 ± 20.8	36.9 ± 26.8	22.3 ± 18.6	Ns	Ns
Red blood cells (10^6^/*μ*L)	4.6 ± 0.5	4.4 ± 0.5	4.6 ± 0.5	4.4 ± 0.5	Ns	Ns
Hematocrit (%)	41.8 ± 4.8	39.8 ± 4.0	41.7 ± 4.6	39.4 ± 4.8	Ns	Ns
Hemoglobin (g/dL)	14.0 ± 1.6	13.4 ± 1.3	14.0 ± 1.5	13.2 ± 1.4	Ns	Ns
MCV (fL)	90.7 ± 5.8	89.7 ± 5.6	91.1 ± 6.1	89.5 ± 6.5	Ns	Ns
MCH (pg)	30.6 ± 2.3	30.0 ± 2.0	30.7 ± 2.1	29.9 ± 2.4	Ns	Ns
White blood cells (10^3^/μL)	6.8 ± 3.5	6.3 ± 1.8	6.7 ± 4.3	6.4 ± 2.1	Ns	Ns
Neutrophils (10^3^/μL)	3.9 ± 1.3	3.7 ± 1.4	3.9 ± 1.3	3.7 ± 1.3	Ns	Ns
Eosinophils (10^3^/μL)	0.3 ± 0.3	0.2 ± 0.2	0.3 ± 0.3	0.2 ± 0.2	Ns	Ns
Basophils (10^3^/μL)	0.05 ± 0.08	0.05 ± 0.05	0.04 ± 0.04	0.04 ± 0.06	Ns	Ns
Monocytes (10^3^/μL)	0.5 ± 0.2	0.4 ± 0.2	0.5 ± 0.2	0.5 ± 0.2	Ns	Ns
Lymphocytes (10^3^/μL)	2.0 ± 0.8	2.1 ± 0.9	2.0 ± 0.7	2.0 ± 0.8	Ns	Ns
Platelets (10^3^/μL)	208.7 ± 77.0	234.5 ± 67.0	209.8 ± 73.7	232.2 ± 75.9	Ns	Ns
Fe (μg/mL)	98.1 ± 35.3	86.6 ± 28.4	97.4 ± 34.6	88.1 ± 30.8	Ns	Ns
Ferritin (ng/mL)	251.5 ± 220.4	178.5 ± 133.5	255.6 ± 221.4	166.9 ± 150.6	Ns	Ns
Vitamin B_12 _(μg/mL)	274.5 ± 166.3	307.7 ± 449.0	292.4 ± 228.1	305.6 ± 271.8	Ns	Ns
Folic acid (ng/mL)	4.6 ± 4.6	5.2 ± 4.8	4.6 ± 4.2	5.3 ± 5.3	Ns	Ns
eGFR (mL/min/1.73 m^2^)	69.6 ± 16.7	67.8 ± 17.6	69.9 ± 17.6	67.8 ± 17.7	Ns	Ns
CRP (mg/L)	5.0 ± 8.4	4.7 ± 7.3	5.1 ± 9.0	4.6 ± 7.5	Ns	Ns
Uric acid (mg/dL)	5.7 ± 1.5	5.3 ± 1.5	5.8 ± 1.6	5.2 ± 1.5	Ns	Ns
25-(OH)-D_3 _(ng/mL)	42.0 ± 23.1	35.9 ± 20.3	42.0 ± 23.6	36.5 ± 21.1	Ns	Ns
Testosterone (ng/mL)	4.4 ± 2.0	0.3 ± 0.3	4.4 ± 2.2	0.3 ± 0.3	Ns	Ns
NSAIDs (*n* (%))	85 (10.5)	132 (17.3)	183 (10.4)	261 (15.8)	Ns	Ns

Ns: not significant; NSAID: nonsteroidal anti-inflammatory drugs.

**Table 2:** Mean values of blood cell counts according to telomere length class (defined by distribution-derived tertiles).

	Classes of telomere length*			*P*
	I	II	III	
All subjects				
RBC (10^6^/*μ*L)	4.48 ± 0.5	4.55 ± 0.48	4.55 ± 0.53	0.08
Hemoglobin (g/dL)	13.60 ± 1.47	13.72 ± 1.45	13.65 ± 1.54	0.7
MCV (fL)	90.26 ± 6.14	89.97 ± 5.59	90.38 ± 5.41	0.2
WBC (10^3^/*μ*L)	6.30 ± 1.9	6.67 ± 3.69	6.64 ± 2.65	0.02
Neutrophils (10^3^/*μ*L)	3.69 ± 1.29	3.82 ± 1.44	3.78 ± 1.37	0.5
Lymphocytes (10^3^/*μ*L)	1.99 ± 0.75	2.09 ± 0.89	2.00 ± 0.91	0.09
Monocytes (10^3^/*μ*L)	0.43 ± 0.19	0.45 ± 0.18	0.46 ± 0.21	0.4
PLT (10^3^/*μ*L)	216.8 ± 68.2	221.7 ± 81.1	225.0 ± 70.2	0.07
Men				
RBC (10^6^/*μ*L)	4.55 ± 0.52	4.61 ± 0.53	4.65 ± 0.54	0.1
Hemoglobin (g/dL)	13.93 ± 1.52	14.10 ± 1.55	14.05 ± 1.63	0.6
MCV (fL)	90.93 ± 6.19	90.65 ± 5.75	90.56 ± 5.45	0.7
WBC (10^3^/*μ*L)	6.44 ± 2.1	6.87 ± 4.81	6.96 ± 3.17	0.01
Neutrophils (10^3^/*μ*L)	3.83 ± 1.25	3.87 ± 1.48	3.94 ± 1.26	0.4
Lymphocytes (10^3^/*μ*L)	1.96 ± 0.78	2.00 ± 0.98	2.02 ± 0.72	0.6
Monocytes (10^3^/*μ*L)	0.47 ± 0.21	0.45 ± 0.16	0.50 ± 0.23	0.5
PLT (10^3^/*μ*L)	203.3 ± 64.3	209.8 ± 92.9	212.9 ± 70.7	0.1
Women				
RBC (10^6^/*μ*L)	4.40 ± 0.46	4.47 ± 0.44	4.44 ± 0.47	0.1
Hemoglobin (g/dL)	13.20 ± 1.31	13.31 ± 1.20	13.26 ± 1.33	0.8
MCV (fL)	89.45 ± 6.03	89.46 ± 5.35	90.09 ± 5.36	0.4
WBC (10^3^/*μ*L)	6.15 ± 1.67	6.39 ± 1.86	6.36 ± 1.87	0.2
Neutrophils (10^3^/*μ*L)	3.55 ± 1.32	3.67 ± 1.39	3.71 ± 1.47	0.5
Lymphocytes (10^3^/*μ*L)	2.02 ± 0.71	2.16 ± 0.78	2.01 ± 1.08	0.01
Monocytes (10^3^/*μ*L)	0.40 ± 0.16	0.46 ± 0.20	0.40 ± 0.18	0.05
PLT (10^3^/*μ*L)	232.0 ± 69.1	233.3 ± 64.7	238.1 ± 67.2	0.5

*Lower and uper limits of TL classes.

All subjects: class I: 1.49–49.38; class II: 49.39–109.00; class III: 109.10–1281.

Men: class I: 1.49–48.04; class II: 48.09–99.99; class III: 100.00–1281.

Women: class I: 1.55–55.15; class II: 50.30–114.40; class III: 116.40–1281.

**Table 3:** Association of blood cells levels, hemoglobin concentration, and MCV with TL according to gender (the table shows coefficients “*r*” and their “*P* value”).

	All subjects	Men	Women
White blood cells (10^3^/*μ*L)	*r* = 0.08	*r* = 0.11	*r* = 0.05
	*P *=* *0.002	*P *=* *0.001	*P* = 0.1
Neutrophils (10^3^/*μ*L)	*r* = 0.03	*r* = 0.04	*r* = 0.03
	*P* = 0.3	*P* = 0.3	*P* = 0.5
Monocytes (10^3^/*μ*L)	*r* = 0.04	*r* = 0.05	*r* = 0.02
	*P* = 0.3	*P* = 0.3	*P* = 0.6
Eosinophils (10^3^/*μ*L)	*r* = 0.007	*r* = 0.04	*r* = −0.03
	*P* = 0.8	*P* = 0.5	*P* = 0.6
Basophils (10^3^/*μ*L)	*r* = 0.14	*r* = 0.16	*r* = 0.11
	*P *=* *0.008	*P *=* *0.04	*P* = 0.1
Lymphocytes (10^3^/*μ*L)	*r* = −0.01	*r* = 0.03	*r* = −0.06
	*P* = 0.6	*P* = 0.4	*P* = 0.1
Red blood cells (10^6^/*μ*L)	*r* = 0.06	*r* = 0.08	*r* = 0.05
	*P *=* *0.02	*P *=* *0.02	*P* = 0.1
Hemoglobin (g/dL)	*r* = 0.01	*r* = 0.02	*r* = 0.01
	*P* = 0.6	*P* = 0.4	*P* = 0.8
MCV (fL)	*r* = 0.001	*r* = −0.01	*r* = 0.02
	*P* = 0.9	*P* = 0.7	*P* = 0.5
Platelets (10^3^/*μ*L)	*r* = 0.06	*r* = 0.06	*r* = 0.04
	*P *=* *0.01	*P* = 0.08	*P* = 0.2

**Table 4:** Correlations between telomere length and red blood count (RBC), white blood count (WBC), and platelet count (PLT): results of multivariate analysis (linear regression model). *R* ^2^ = 0.16 for RBC model, *R* ^2^ = 0.07 for WBC model, and *R* ^2^ = 0.06 for PLT model.

Independent variable	RBC		WBC		PLT	
	*β* ± SD	*P*	*β* ± SD	*P*	*β* ± SD	*P*
Male gender	−0.001 ± 0.046	0.98	−0.873 ± 0.210	<0.001	29.358 ± 7.537	<0.001
Age (years)	−0.012 ± 0.002	<0.001	−0.020 ± 0.008	0.010	−3.218 ± 1.443	0.02
Vitamin B_12 _(*μ*g/mL)	0.001 ± 0.000	0.08	0.001 ± 0.000	0.5	0.001 ± 0.006	0.8
Folic acid (ng/mL)	−0.008 ± 0.003	0.004	−0.028 ± 0.012	0.02	0.225 ± 0.440	0.6
Fe (*μ*g/mL)	0.001 ± 0.000	0.005	−0.007 ± 0.002	<0.001	−0.163 ± 0.071	0.02
CRP (mg/L)	−0.003 ± 0.002	0.08	0.043 ± 0.008	<0.001	1.171 ± 0.268	<0.001
eGFR (mL/min/1.73 m^2^)	0.003 ± 0.001	<0.001	−0.011 ± 0.004	0.004	0.258 ± 0.136	0.06
Testosteron (ng/mL)	0.040 ± 0.009	<0.001	−0.081 ± 0.041	0.05	1.346 ± 1.485	0.4
NSAIDs (yes)	−0.009 ± 0.036	0.80	−0.116 ± 0.165	0.5	−2.160 ± 5.900	0.7
Telomere length*	0.029 ± 0.013	0.03	0.154 ± 0.062	0.01	2.380 ± 2.222	0.3

*Variable logarithmically transformed.
